# Periodic Nocturnal Cough in Infants

**Published:** 1892-12

**Authors:** 


PERIODIC NOCTURNAL COUGH IN INFANTS.—(Traite des Maladies des Enfants. *Journ. de Med. et de Chir. Prat.,* 1892, lxiii, 5, p. 192; *Archives of Gynœcology,* July, 1892, p. 341.) By Baginsky.

Baginsky describes a nocturnal cough of obscure origin in infants. Without apparent cause the patient is awakened by violent cough lasting from a quarter to half an hour. At the end of the paroxysm the child again goes to sleep, and is well in the morning. Ordinarily physical examination shows nothing abnormal. Such attacks have been ascribed to malaria, but this cause can often be excluded. Most probably the condition is due to sub-acute or chronic rhino-pharyngitis or to a bronchial catarrh with hyperæsthesia of the mucous membrane of the respiratory tract, the accumulation of mucus during sleep causing irritation and thus inducing the paroxysm.—*Int. Med. Mag.*

